# Advancing oral immunology for improving oral health

**DOI:** 10.1007/s00056-023-00473-3

**Published:** 2023-06-14

**Authors:** James Deschner, Agnes Schröder, Manuel Weber, Kerstin Galler, Peter Proff, Christian Kirschneck, Aline Bozec, Jonathan Jantsch

**Affiliations:** 1 Department of Periodontology and Operative Dentistry, University Medical Center Mainz, Augustusplatz 2, 55131 Mainz, Germany; 2 Department of Orthodontics, Universitätsklinikum Regensburg, Franz-Josef-Strauß-Allee 11, 93053 Regensburg, Germany; 3 Institute of Clinical Microbiology and Hygiene, Universitätsklinikum Regensburg, Franz-Josef-Strauß-Allee 11, 93053 Regensburg, Germany; 4 Department of Oral and Cranio-Maxillofacial Surgery, Universitätsklinikum Erlangen, Glückstr. 11, 91054 Erlangen, Germany; 5 Department of Conservative Dentistry and Periodontology, Universitätsklinikum Erlangen, Glückstr. 11, 91054 Erlangen, Germany; 6 Department of Medicine 3, Rheumatology and Immunology, Universitätsklinikum Erlangen, Glückstr. 6, 91054 Erlangen, Germany; 7 Institute for Medical Microbiology, Immunology and Hygiene and Center for Molecular Medicine Cologne (CMMC), University Hospital Cologne and Faculty of Medicine, University of Cologne, Goldenfelsstr. 19–21, 50935 Cologne, Germany

**Keywords:** Immune system, Oral diseases, Oral microbiota, Immune responses, Host microbial interactions, Immunsystem, Orale Erkrankungen, Orale Mikrobiota, Immunreaktionen, Mikrobiom-Wirt-Interaktionen

## Abstract

Although substantial progress has been made in dentistry in terms of diagnosis and therapy, current treatment methods in periodontology, orthodontics, endodontics, and oral and maxillofacial surgery, nevertheless, suffer from numerous limitations, some of which are associated with a dramatic reduction in the quality of life. Many general mechanisms of inflammation and immunity also apply to the oral cavity and oral diseases. Nonetheless, there are special features here that are attributable, on the one hand, to developmental biology and, on the other hand, to the specific anatomical situation, which is characterized by a close spatial relationship of soft and hard tissues, exposure to oral microbiota, and to a rapid changing external environment. Currently, a comprehensive and overarching understanding is lacking about how the immune system functions in oral tissues (oral immunology) and how oral immune responses contribute to oral health and disease. Since advances in translational immunology have created a game-changing shift in therapy in rheumatology, allergic diseases, inflammatory bowel disease, and oncology in recent years, it is reasonable to assume that a better understanding of oral immunology might lead to practice-changing diagnostic procedures and therapies in dentistry and thereby also profoundly improve oral health in general.

## Abstract (de)

Obwohl in der Zahnmedizin in Bezug auf Diagnose und Therapie wesentliche Fortschritte erzielt wurden, weisen die derzeitigen Behandlungsmethoden in Parodontologie, Kieferorthopädie, Endodontie sowie Mund‑, Kiefer- und Gesichtschirurgie zahlreiche Einschränkungen auf, von denen einige mit einer dramatischen Beeinträchtigung der Lebensqualität verbunden sind. Zahlreiche grundlegende Prozesse von Entzündung und Immunität gelten auch für die Mundhöhle und orale Erkrankungen. Dennoch gibt es hier Besonderheiten, die zum einen auf entwicklungsbiologische Faktoren, zum anderen auf die spezifische anatomische Situation zurückzuführen sind. Diese ist durch ein enges räumliches Verhältnis von Weich- und Hartgewebe, die Exposition gegenüber der oralen Mikrobiota und eine sich schnell verändernde äußere Umgebung gekennzeichnet. Derzeit mangelt es an einem umfassenden und übergreifenden Verständnis der Funktionen des Immunsystems in oralen Geweben (orale Immunologie) und der Frage, wie orale Immunreaktionen zu oraler Gesundheit bzw. Krankheit beitragen. Da in den vergangenen Jahren Fortschritte in der translationalen Immunologie zu einem Paradigmenwechsel in der Rheumatologie, bei allergischen Erkrankungen, entzündlichen Darmerkrankungen und in der Onkologie geführt haben, liegt die Vermutung nahe, dass ein besseres Verständnis der oralen Immunologie zu praxiswirksamen Diagnoseverfahren und Therapien in der Zahnmedizin führen und damit auch die Mundgesundheit im Allgemeinen grundlegend verbessern könnte.

## Introduction

The immune system plays a central role in the organism to maintain tissue integrity and functionality. To this end, it mediates adaptation to various endogenous and exogenous stressors. For example, the immune system plays a paramount role in the defense against infections and in promoting wound healing [1]. Recent findings show that the immune system also takes on a very important supporting and accessory role in maintaining the physiological function of numerous organ systems (immunophysiology) [2, 3].

Unfortunately, the results of immune reactions are not always beneficial. The immune system can also maintain and further fuel pathological processes through misguided activation and perpetuation of detrimental immune reactions ultimately resulting in tissue destruction (immunopathology). Therefore, research into molecular mechanisms that promote resolution of inflammation plays a very important role, especially in rheumatic diseases, inflammatory bowel diseases, and allergic diseases [4]. Nevertheless, there are even concepts in infection immunology proposing the promotion of tolerogenic immune responses for the treatment of infections [5]. Immunotherapy is, therefore, an important treatment modality in many (if not all) medical specialties.

Although many general mechanisms of inflammation and immunity also apply to the oral cavity, there are special features here that are attributable, on the one hand, to developmental biology and, on the other hand, to the specific anatomical situation, which is characterized by a close relationship of soft and hard tissues, constant exposure to microbiota and rapid changes in environmental factors [6]. In dentistry, immunological research has largely focused on periodontal diseases. We are convinced that the study of immunology in all areas of dentistry (oral immunology) holds great potential to foster oral health not only in periodontal diseases, but also in orthodontics, endodontics, and oral and maxillofacial surgery. This is significant because oral diseases are very prevalent worldwide and significantly affect quality of life [7] and current strategies in dentistry have not succeeded in solving the problems [8].

## Periodontal diseases

Periodontal diseases, i.e., diseases of the tooth-supporting tissues (periodontium), are among the most common human diseases of all [9]. In particular, periodontitis, which is characterized by irreversible bone and attachment loss as well as tooth loss, still poses major problems for dental practitioners. A large number of publications have shown that periodontitis is associated with numerous diseases of the whole organism, such as diabetes mellitus, cardiovascular diseases, and rheumatoid arthritis [10, 11]. Even though the prevalence of dental caries has been successfully reduced by preventive measures in recent decades, the prevalence of periodontitis is still high, partly due to an aging society [12, 13].

Although it is accepted that the interplay of the oral microbiome and oral mucosal immune responses plays an important role in periodontitis and subsequent systemic inflammatory conditions [14], a comprehensive picture on the role of the immune system in the etiopathogenesis and therapy of oral diseases in general is still missing. In particular, the interplay between the oral tissue microenvironment and the immune response at the molecular and cellular levels is not yet fully understood.

In periodontitis and peri-implantitis [15], the host immunoinflammatory response is largely responsible for the course and extent of the diseases [11]. In health, resident polymicrobial communities maintain an ecological balance on mucosal surfaces. In doing so, they engage in both intermicrobial and host–microbial interactions. However, genetic and acquired destabilizing factors can destroy this homeostatic equilibrium, leading to selection of periodontally destructive species [16, 17]. This process is called dysbiosis and is typical of periodontitis [17]. Periodontitis is epidemiologically associated with other chronic inflammation-related diseases, for example, cardiometabolic, neurodegenerative, and autoimmune diseases. That these are causal relationships is evidenced by clinical therapy intervention studies and animal studies [11]. As mentioned above, periodontitis results from mutually reinforcing interactions between a dysbiotic microbiome and dysregulated inflammation. Inflammation is not only a consequence of dysbiosis, but also promotes further growth of selectively dysbiotic bacterial communities (inflammophilic bacteria) by mediating tissue dysfunction and damage, thereby perpetuating the disease [18]. This raises the possibility of developing host modulation therapies in the treatment of periodontitis and then later using them clinically. Host modulation could inhibit tissue destructive inflammatory processes and promote their resolution or directly interfere with connective tissue and bone destruction [19]. In line with this, targeted approaches to modify host immune responses could be attractive novel approaches to treat periodontal disease [20]. For example, as reviewed elsewhere, the complement system has been shown to be hyperactivated in periodontitis and therefore its inhibition is therapeutically promising [21]. Thus, by disrupting the complement cascade at its central component, C3, with a locally administered small peptide compound (Cp40/AMY-101), nonhuman primates could be protected from periodontitis [21]. Consequently, a C3-targeted intervention may have future application as a complementary treatment for periodontal disease in humans [22]. Once-weekly intragingival injection of this small peptide compound in humans over a 3-week period was safe and resulted in significant reduction of tissue-destructive proteases [23]. Nonetheless, immunomodulatory therapies for periodontitis and peri-implantitis are not standard of care [24]. These approaches are still in their infancy and require comprehensive basic science and translational studies of, ideally, all cellular and molecular players involved in the inflammation-driven tissue-destructive processes. In peri-implantitis, this might ultimately lead to, for instance, bioactive immune-modulating surfaces and treatments [1, 5, 25].

## Endodontic diseases

In endodontics, most current procedures clinically still end with the removal of pulp tissue instead of guiding endodontic immunoinflammatory processes. Successful elimination of microbial infections in the root canal remains a major challenge. Currently, antimicrobial drugs are used in endodontic therapy, some of which are ineffective due to antimicrobial resistance. In addition, the interaction of these agents with the immune system can be suppressive or stimulatory. Non-conventional antimicrobial drugs, such as antimicrobial peptides, propolis and nanomaterials, sometimes have a strong antimicrobial effect and at the same time exert immunomodulatory and/or reparative effects [26]. For example, the antimicrobial peptide LL-37 plays an important role in odontoblast cell differentiation, in reparative dentin formation and provision of signals for defense by activating the innate immune system [27]. Antimicrobial peptides and their synthetic mimics, in addition to their antimicrobial effects, possess bioactive functions such as immunomodulation and improvement of wound healing, so that in the future they could be used as adjuvants in the treatment of caries, infected pulp and root canals [28]. The dentin–pulp complex can recognize invading bacteria during infection [29]. Odontoblasts, fibroblasts, stem cells, endothelial cells, and immune cells react to the bacteria, their components, and products. Controlling and eliminating infection is certainly key for repairing dental tissues. Nonetheless, in the future, anti-inflammatory and immunomodulatory approaches based on molecular, epigenetic, and photobiomodulatory technologies could revolutionize endodontic therapy [30].

## Orthodontic tooth movement

Orthodontic tooth movement is often associated with adverse side effects due to the incomplete understanding of involved immunoinflammatory tissue reactions. During orthodontic tooth movement, remodeling of the periodontal ligament and alveolar bone occurs. In response to the orthodontic forces, inflammatory processes develop in terms of a sterile inflammatory reaction [31], which on the one hand enable movement of mispositioned teeth through the alveolar bone to their physiological position; on the other hand, these inflammatory processes may also lead to dental root resorption as an undesirable side effect in orthodontic therapy [32]. A better understanding of immunoinflammatory processes during orthodontic treatment could lead to prevention or at least reduction of root resorptions during orthodontic treatment. This knowledge could also be used to accelerate orthodontic therapies, as it is known that the velocity of tooth movement is directly dependent on the remodeling activity of alveolar bone, which is mitigated by the immunoinflammatory reactions triggered by applying a mechanical force to teeth for the purpose of treatment [33, 34]. Although mechanical stress had been shown to induce inflammation in orthodontic tooth movement over two decades ago [32, 35], in immunology, mechanotransduction has only recently received widespread attention as an important regulator of immune responses [36]. The field of orthodontic research is certainly the perfect area to explore these concepts and to advance our understanding of osteoimmunology [37].

## Maxillofacial diseases

Bisphosphonates are commonly prescribed antiresorptive drugs by several medical specialties. They inhibit osteoclast function and therefore prevent bone resorption [38]. This antiresorptive capability is utilized in management of osteoporosis and in treatment of bone metastases caused by malignant tumors. However, recent data indicate an anti-tumoral effect of bisphosphonates beyond growth inhibition of bone metastases [39]. This is attributed to immune modulatory effects of bisphosphonates [40]. However, in maxillofacial tissues, bisphosphonates and other antiresorptive drugs can cause severe side effects. Medication-associated osteonecrosis of the jaw (MRONJ) with necrosis of bone, soft tissue, and consecutive loss of teeth is the most severe and dreaded side effect of bisphosphonates [41]. The restriction of this side effect to maxillofacial tissues as well as the exact pathophysiology is not yet known [41, 42] and needs to be further analyzed. Transcortical blood vessels constitute the main blood supply to long bones and are remodeled by osteoclasts [43]. Whether bisphosphonate-mediated disturbances in immunovascular function underlie MRONJ is unclear and requires investigation.

Systemic immunosuppression is known to influence oral health. Especially, the immunosuppressive calcineurin inhibitors, such as cyclosporin A, are characterized by a major side effect of gingival overgrowth. The pathophysiological mechanisms underlying this phenomenon are still unknown, but it is very likely that distorted immune responses play a key role, which warrants detailed investigation [44].

Finally, inflammation in maxillofacial tissues is also linked to the development of oral malignancies. For instance, there is an association of *Streptococcus mutans* infection with the development of oral cancer [45]. Moreover, local immunologic changes precede the development of oral cancer in pre-existing oral leukoplakia. For instance, there is an association of oral cancer formation with macrophage polarization [46] and with the expression of programmed cell death ligand 1 and its receptor, which are important inhibitory regulators of immune responses [47]. Therefore, modification of oral immunity holds great potential in preventing malignant transformation of oral leukoplakia.

## Conclusion

The findings show that complex interactions take place in, on, and around the teeth between the oral microbiome, immune cells, and oral tissue-resident cells. A better understanding of the oral microbiome, oral immune responses and oral tissues, and the interactions between and among them will help to develop novel biomarkers and new immune-based procedures for the therapy of periodontal, endodontic and orthodontic (i.e., dental) and maxillofacial diseases. Without a shadow of a doubt, this holds great potential to fundamentally transform clinical practice in dentistry in general.
